# The First Food of Infants

**Published:** 1856-04

**Authors:** Frederick J. Brown


### THE FIRST FOOD OF INFANCY.

By FREDERICK J. BROWN, M.D.

One means of improving the human species consists in substituting good cow's milk for the breast-milk, in all cases in which there is ill health on the part of the mother, or when an hereditary predisposition to disease exists. Children brought up by hand suffer from thrush, and pine away, in many instances; but this is the consequence of their being fed with biscuit, gruel, and other articles.

If cow's milk be mixed with a small proportion of water or barley-water, and white sugar, and if it be then gently warmed and introduced into a suck-bottle, it will be readily taken by an infant, both by day and by night. Nothing else is required until the child arrives at the age of three or four months, when it may be fed with plainly boiled or scalded white bread, with a small quantity of white sugar, in addition to the cow's milk given by the suck-bottle. Children, when thus brought up, thrive and escape disorder of the digestion. I am acquainted with a family that is afflicted, both hereditarily and personally, with scrofula, gout, spinal curvature, and insanity. There were eight children. The youngest two are dead. All were suckled by the mother, excepting the fifth; and that one is strikingly different, both in mental powers and physical conformation, from the rest of the family. He is tall, straight, and strong, and is possessed of good abilities. So much for cow's milk *versus* tainted breast-milk.

Where the mother is free from both hereditary predisposition and actual disease, and when she is sufficiently strong to perform the function, there can be no doubt that child-suckling is desirable. It accords with the natural maternal instinct, and it serves the wise purpose of increasing the attachment that exists between the mother and her offspring; and it may, possibly, communicate to the child some of the mother's qualities, as well mental as physical.

The normal duration of lactation is a subject that claims attention. It would be well to ascertain what it is in the mammalia throughout the series. Some principle common to the class will probably be discovered, namely:—

Duration of lactation in some ratio with utero-gestation; or in proportion to the condition of the masticatory apparatus; or in relation with the capability of the young animal to provide for its own sustenance.

Whilst these inquiries are going forward, it is advisable to adopt some rule in practice. The following is the rule recommended for general adoption :—Nine months to bear the child, and nine months to suckle it.

This rule should be earnestly impressed upon the maternal mind. Women are continually applying for advice for dyspeptic and nervous disorders that result most markedly from over-suckling. Succeeding children must be weakened in constitution by the injury to the mother's health induced by over-suckling. In no case should the mother suckle beyond nine months ; and when symptoms of super lactation manifest themselves at an earlier period than nine months, no matter how early the period, the mother should abandon suckling.

Lactation should be desisted from whenever the catamenia appear ; no matter how soon, excepting only the first after parturition, which is natural. The system cannot support with impunity the concurrence of these two secretions.

One word as to weaning : there is no necessity to disgust the child with the mother's breast. Give it the suck-bottle and withhold the breast. The mother may use purgatives to relieve the breasts. The child may suck the bottle as long as it pleases. This method of weaning removes from the mother's mind the idea of harshness.

It is to be hoped that no woman will ruin her own health and weaken the constitution of her future offspring under the idea of escaping from conception during lactation. Conception rarely occurs during the normal period of lactation, but it is not prevented by a prolongation of the period. In those cases in which pregnancy occurs during the early months of lactation, there should be the immediate abandonment of suckling. All women should practise this rule.

Chatham, Kent, March 1856.

[We have placed the useful papers of Drs. Barker and Brown side by side, because it is at once instructive, and confirmatory of truth, to find two independent observers taking such sound and common-sense views on one of the most important, though most neglected, subjects connected with sanitary science. The views maintained by these gentlemen will be readily accepted by the profession ; but it is to the public generally that such teachings most forcibly appeal. EDITOR.]

